# Genetic Characterization of Huacaya Alpacas (*Vicugna pacos*) in Poland Using Microsatellite Markers

**DOI:** 10.3390/biology15171555

**Published:** 2026-09-05

**Authors:** Angelika Mąsior, Karolina Zygmunt

**Affiliations:** Department of Animal Molecular Biology, National Research Institute of Animal Production, Krakowska 1, 32-083 Balice, Poland; karolina.zygmunt@iz.edu.pl

**Keywords:** alpacas, individual identification, sex identification marker, parentage testing, genetic diversity, population structure

## Abstract

Alpacas (*Vicugna pacos*) are of increasing economic importance, mainly due to their high-quality fiber. This study aimed to develop and validate molecular tools for sex identification, individual identification and parentage testing, and to characterize genetic diversity and population structure in Huacaya alpacas bred in Poland. Forty-five previously described microsatellites and one newly developed SRY-based marker were organized into three multiplex panels containing sixteen, seventeen and thirteen markers. The SRY marker was validated in 17 reference animals of known sex (5 males, 12 females) and subsequently in 239 study animals, showing complete concordance with recorded sex. The 44 microsatellites, excluding SRY and the monomorphic YWLL02, showed a mean of 11.45 alleles per locus, observed heterozygosity (Ho) of 0.708, expected heterozygosity (He) of 0.755 and mean polymorphism information content (PIC) of 0.727. Combined non-exclusion probabilities were 1.58 × 10^−11^ for a first parent and 1.08 × 10^−30^ for a parent pair. Seven loci showing elevated null allele frequencies were excluded from population structure analyses. Multivariate, phylogenetic and Bayesian analyses revealed no strong genetic subdivision. The developed tools provide practical support for genetic management of alpacas in Poland.

## Simple Summary

Alpacas are increasingly bred worldwide because of their valuable fiber, but maintaining genetic diversity is essential for the long-term health and adaptability of their populations. In Poland, alpaca breeding is relatively recent, and genetic studies are important for monitoring population development and supporting responsible breeding practices. Alpacas occur in two phenotypic varieties, Suri and Huacaya. In this study, we developed and evaluated molecular tools for Huacaya alpacas bred in Poland, including a genetic marker for sex identification and microsatellite marker panels used for individual identification and parentage verification. The developed tools showed high accuracy and can be applied in breeding programs and genetic monitoring. The Polish Huacaya alpaca population showed high genetic diversity and no signs of substantial inbreeding, indicating that current breeding practices have maintained a broad genetic background. Genetic analyses also revealed a relatively uniform population structure, without clear division into separate genetic groups. These findings provide important information for the management and conservation of alpaca genetic resources in Poland and demonstrate the usefulness of molecular markers in supporting sustainable breeding strategies.

## 1. Introduction

Alpacas (*Vicugna pacos*) belong to the domesticated South American camelids (SACs), and their native habitat is the Andean Altiplano region. They are closely related to another domesticated species, the llama (*Lama glama*), as well as to two wild species: the vicuña (*Vicugna vicugna*) and the guanaco (*Lama guanicoe*). All four species belong to the tribe *Lamini* within the family *Camelidae* and share the same diploid chromosome number (2*n* = 74) [1].

Global interest in alpaca farming is primarily driven by the high value of the fiber obtained from these animals. The quality and characteristics of alpaca fleece are influenced by both genetic and environmental factors. Owing to their considerable adaptive capacity, largely resulting from physiological resilience to diverse climatic conditions, alpacas are currently bred in many regions worldwide [2].

In Poland, alpaca breeding was initiated in 2004 and remains sufficiently competitive to sustain a steadily increasing level of interest [3,4]. Initially, 100 alpacas were imported from Chile; however, the animals arrived without pedigree documentation [5]. They constituted two herds carefully selected by the Chilean company Quintessence Alpaca International [6]. The proportion of females and males was not disclosed, but it can be assumed that the majority were females. Due to the lack of pedigree information, it is not possible to determine whether the animals were related. Nevertheless, they had been selected to establish a new breeding herd (Alpaki w Polsce; Mariusz Wierzbicki) [7]. Furthermore, a study conducted in 2021 on the Polish alpaca population revealed no evidence of inbreeding [4]. Therefore, it may be assumed that the animals were not closely related. However, it should be noted that the initial group of 100 alpacas was not assessed for inbreeding.

A somewhat misleading perception persists that alpacas are easy to manage and have minimal requirements with regard to nutrition and husbandry. As recently as a decade ago, alpacas were regarded as exotic animals in Poland. Since that time, the level of knowledge among breeders and veterinary professionals has expanded substantially, a process that required considerable determination and commitment from the communities involved in alpaca husbandry and healthcare [8]. Within the country, alpacas are kept mainly for fiber production, but they are also employed in animal-assisted interventions. Specifically, alpaca-assisted therapy (alpacotherapy) is used to support both physical and cognitive rehabilitation in individuals with disabilities [9,10].

Large-scale studies on the genetic diversity and population structure of alpacas have been conducted primarily in South American countries that maintain the largest alpaca populations. Such assessments have been performed repeatedly in Peru [11,12,13,14] and Bolivia [15,16]. Nevertheless, genetic research on alpacas has not yet been carried out on a scale comparable to that of other livestock species. To date, genetic studies of alpacas conducted in Poland have focused on the identification of alpaca × llama hybrids as well as on the preliminary assessment of the genetic diversity of alpaca populations. For this purpose, analyses have employed 17 microsatellite markers recommended by the International Society for Animal Genetics (ISAG) [4], along with mitochondrial DNA (mtDNA) and the Y-chromosome gene *DBY* [17].

Additionally, since 2022, the Laboratory of Molecular Genetics of the National Research Institute of Animal Production (Poland) has served as the Duty Laboratory for the ISAG International Llama/Alpaca STR DNA Typing Comparison Test [8]. These Comparison Tests are organized biennially and enable the establishment of standardized allele nomenclature across participating laboratories. Consequently, DNA profiles generated by laboratories that achieve rank I are internationally comparable and can be used for parentage testing across laboratories.

During the 2022/2023 Comparison Test, alleles with extreme fragment sizes (bp) were identified in 2 of the 22 analyzed samples. In one sample, an unusual allele (186 bp) was detected at the LCA37 locus; however, this allele was not identified by some participating laboratories. A similar issue was observed in another sample at the LCA19 locus, where an allele with an extreme fragment size (136 bp) was detected. In both cases, these alleles overlapped with the size ranges of amplification products from other markers, thereby increasing the risk of their being overlooked [18].

The aim of this study was to modify the previously used STR marker panel for alpacas to overcome the issue of allele overlap in future analyses. Additionally, a genetic marker for sex identification was developed and incorporated into one of the proposed panels. Three microsatellite panels were developed and evaluated for their suitability for individual identification and parentage verification. These panels were subsequently applied to assess the genetic diversity and population structure of Huacaya alpacas bred in Poland.

## 2. Materials and Methods

### 2.1. Sample Collection

DNA extracts obtained from 239 Huacaya alpacas were used in the analysis. The DNA originated from biological material submitted to the laboratory between 2023 and 2026 as part of routine procedures for establishing DNA profiles of animals. The initial dataset comprised 742 alpacas. However, due to missing alleles at one or more STR loci in some individuals, only animals with complete genotype profiles for all analyzed STR markers were included in the final dataset. As a result, 239 animals were included in the analysis. The analyzed population may include animals from different generations, as well as parent–offspring relationships, full siblings, half-siblings, or other types of relatedness. Therefore, the analyzed dataset should not be considered a set of unrelated individuals or founder animals. The 239 animals represent a random subset of individuals with complete STR profiles from the available population of 742 alpacas. Additionally, 17 DNA isolates obtained from the Alpaca/Llama Comparison Test 2022/2023 were used in the development of a proprietary marker for sex identification in alpacas [8].

### 2.2. Sex Identification Marker

Seventeen of the twenty-two samples were analyzed, all originating from the Llama/Alpaca Comparison Test 2022/2023 (the remaining five samples were identified as llamas) [8]. The sex of the 17 alpacas was known, including 5 males and 12 females. A 116-base-pair fragment of the male alpaca SRY gene was identified in the NCBI (National Center for Biotechnology Information) database (EF563984.1). Primers (5′–3′) specific to the identified fragment were designed using Primer3 [19,20,21]: GGCTCTAGAGATCCCAAAATGC and TAGTCTCTGTGCCTCCTCGA.

Based on the length of the SRY gene fragment, one of the primers, TAGTCTCTGTGCCTCCTCGA, was labeled with the fluorescent dye PET (Thermo Fisher, Scientific, Waltham, MA, USA), so that, in the event of successful amplification, it could be included in multiplex panel I.

Initially, the primers were tested in a singleplex PCR reaction to confirm the generation of the expected PCR product. The reaction mixture contained 10.25 μL of Type-it Microsatellite PCR Kit (Qiagen, GmbH, Hilden, Germany), 1.25 μL of primer, and 1 μL of DNA (10 ng/μL). DNA concentration and quality were assessed using a NanoDrop (Thermo Fisher, Scientific, Waltham, MA, USA). DNA amplification was performed using the thermal cycling program presented in Table S1. Following successful verification, the primers were incorporated into multiplex panel I.

Capillary electrophoresis was performed using a 3500xl Genetic Analyser (Applied Biosystems, Foster City, CA, USA) and contained 11 μL of Hi-Di™ Formamide (Thermo Fisher, Scientific, Waltham, MA, USA), 0.3 μL of GeneScan™ 500 LIZ™ dye Size Standard (Thermo Fisher, Scientific, Waltham, MA, USA), and 1 μL of PCR product. Samples were denatured for 5 min at 95 °C. The resulting genotype calling was carried out using GeneMapper^®^ Software 5.0 (Applied Biosystems, Foster City, CA, USA).

### 2.3. Microsatellite Genotyping

A total of 45 markers described in previous scientific studies and 1 marker SRY were used (Table 1). Some of them were recommended by ISAG [22]. In total, three multiplex PCR reactions were prepared. The panels were prepared and optimized for multiplex reactions. The first panel consisted of 16 markers, the second of 17, and the third of 13 (Table 1). Amplification was performed using a VeritiPro Thermo Cycler (Applied Biosystems, Foster City, CA, USA). The thermal cycling program listed in Table S1 was used for the multiplex PCR of all three panels. The reaction mixture contained 10.25 μL of Type-it Microsatellite PCR Kit, 1.25 μL of primer mix, and 1 μL of DNA (30 ng/μL). Capillary electrophoresis and genotyping are described in Section 2.2.

### 2.4. Bioinformatic Analysis

Bioinformatics analyses were performed to assess genetic diversity, conduct parentage testing, and characterize population structure using various software packages and tools, including GenAlEx [33], Cervus [34], Python 3.13.3 (with the following packages: NumPy 2.2.5, SciPy 1.15.3, Matplotlib 3.10.3, Biopython 1.87, and Pillow 11.2.1), and STRUCTURE software [35]. Bayesian clustering analysis implemented in STRUCTURE was conducted using a burn-in period of 200,000 iterations, followed by 500,000 Markov chain Monte Carlo (MCMC) repetitions. The number of assumed clusters (K) ranged from 1 to 10, with 10 independent runs performed for each K value. StructureSelector [36] was used to determine the most likely number of clusters based on Evanno’s ΔK method [37] while CLUMPAK was used for result summarization and graphical visualization [38]. Individual differentiation was visualized using principal coordinates analysis (PCoA) and presented as a heatmap of pairwise genetic distances, with individuals ordered according to hierarchical clustering using the UPGMA method implemented in the scipy.cluster.hierarchy module. A genetic distance tree was constructed using the Neighbor-Joining method [39], implemented in Biopython, and visualized as a circular cladogram using iTOL v6 [40]. The composition of the three marker panels and the selection of markers included in the genetic diversity and subsequent population structure analyses are summarized in Table S2.

## 3. Results

### 3.1. Sex Determination Marker

Fragment analysis revealed a peak corresponding to an allele located at the 114-base-pair region. The peak was observed in all male individuals, whereas the allele was absent in females. The localization of the allele allowed the identified region to be incorporated into multiplex panel I as a marker for sex identification in these animals. The obtained results were additionally validated across all individuals included in the genetic diversity assessment conducted for the purposes of this study.

### 3.2. Assessment of Three Microsatellite Panels for Individual Identification and Parentage Testing

Three multiplexes were prepared and then assessed for their suitability for genetic analysis (Table 2, Table 3 and Table 4).

The 14 microsatellite loci (Table 2) (two markers were monomorphic and were therefore excluded from the bioinformatic analysis: SRY and YWLL02) exhibited a high level of genetic variability, with the number of alleles (Na) per locus ranging from 5 to 16 and averaging 11.14 alleles. Mean observed (Ho) and expected heterozygosities (He) were 0.778 and 0.786, respectively, indicating high genetic diversity and only a slight heterozygote deficit. The average polymorphic information content (PIC) was 0.764, demonstrating that the marker panel was highly informative. The most informative locus was LCA65 (PIC = 0.856), whereas LGU91 showed the lowest PIC value (0.572). Mean non-exclusion probabilities were 0.554 for the first parent (NE-1P), 0.402 for the second parent (NE-2P), 0.214 for a parent pair (NE-PP), 0.078 for identity (NE-I), and 0.370 for sibling identity (NE-SI). The low average F(Null) value (0.0072) suggests a negligible influence of null alleles.

The 17 microsatellite loci (Table 3) exhibited a high level of genetic variability, with Na ranging from 2 to 29 and averaging 10.235 alleles. Mean Ho and He were 0.657 and 0.718, respectively, indicating substantial genetic diversity and a slight heterozygote deficit across the studied population. The average PIC was 0.691, confirming that the marker panel is highly informative for genetic analyses. The most informative locus was YWLL08 (PIC = 0.932), whereas YWLL46 showed the lowest PIC value (0.339). Mean non-exclusion probabilities were 0.648 for NE-1P, 0.490 for NE-2P, 0.337 for NE-PP, 0.144 for NE-I, and 0.427 for NE-SI. The average F(Null) value (0.0596) suggests a low to moderate presence of null alleles, with some loci (YWLL59 and CMS3) showing elevated values.

The 13 microsatellite loci (Table 4) exhibited a moderate to high level of genetic variability, with Na ranging from 4 to 23 and averaging 13.385 alleles. Mean Ho and He were 0.700 and 0.772, respectively, indicating substantial genetic diversity in the studied population with a slight heterozygote deficit. The average PIC was 0.739, confirming that the marker set is highly informative for genetic analyses. The worst informative locus was CMS104 (PIC = 0.389), whereas VOLP32 showed the highest PIC value (0.846). Mean non-exclusion probabilities were 0.575 for NE-1P, 0.428 for NE-2P, 0.253 for NE-PP, 0.101 for NE-I, and 0.378 for NE-SI, indicating good overall efficiency for parentage and individual identification analyses. The mean F(Null) value was 0.0522, suggesting a low-to-moderate presence of null alleles, with some loci (e.g., LCA68) showing elevated values that may indicate potential genotyping bias and should be interpreted with caution.

The combined NE-1P indicates that a random individual would have only a 0.02499%, 0.01557%, and 0.04072% chance of not being excluded as a parent for panels I, II, and III, respectively (Tables S3–S5). In other words, the corresponding exclusion probabilities for an incorrect parent were 99.97%, 99.98%, and 99.96%, respectively. The combined NE-2P demonstrated even higher efficiency when one parent was known, with exclusion probabilities exceeding 99.99% for all three panels. The combined NE-PP were 9.908 × 10^−11^, 2.721 × 10^−11^, and 4.012 × 10^−10^ for panels I, II, and III, respectively, indicating that the probability of failing to exclude an unrelated parent pair was virtually zero. Accordingly, the exclusion power exceeded 99.99% in all cases. The combined NE-I were 2.777 × 10^−17^, 1.653 × 10^−18^, and 4.746 × 10^−16^ for panels I, II, and III, respectively, demonstrating a very high capacity for individual identification. These values indicate that the probability of two unrelated individuals sharing an identical genetic profile is negligible. Similarly, the combined NE-SI were 1.02 × 10^−6^, 3.2 × 10^−7^, and 3.47 × 10^−6^ for panels I, II, and III, respectively, confirming the strong discriminatory power of all three panels even among closely related individuals, such as siblings (Tables S3–S5).

Overall, each of the three panels exhibited high levels of polymorphism, substantial heterozygosity, and strong exclusion power, confirming their suitability for genetic diversity studies, individual identification, and parentage verification in Huacaya alpacas. Furthermore, each panel demonstrated sufficient discriminatory power to be effectively applied independently.

The combined NE-P for all 44 markers (Table 5) were exceptionally low and amounted to 1.584 × 10^−11^ for the first parent, 2.720 × 10^−18^ for the second parent, and 1.081 × 10^−30^ for the parent pair. The probability of non-exclusion of individual identity reached 2.178 × 10^−50^, while for siblings it was 1.125 × 10^−18^. These results indicate a very high discriminatory power of the analyzed microsatellite marker panel, both for individual identification and parentage testing.

### 3.3. Genetic Diversity of Huacaya Alpacas

Using three prepared panels, the genetic diversity of Huacaya alpacas bred in Poland was analyzed. The SRY marker (sex identification) and YWLL02 (monomorphic), which were included in Panel I, were excluded from the analysis. In total, 44 out of 46 markers were used. The genetic diversity parameters of Huacaya alpacas are presented in Table 6.

The study population was characterized by high genetic diversity, as indicated by the mean Na = 11.45, a high effective number of alleles (Ne = 5.14), a high Shannon index (I = 1.756), and a high He = 0.755. Ho was slightly lower than expected (0.708), which was reflected in a positive mean inbreeding coefficient (F = 0.0635). Most loci were in Hardy–Weinberg equilibrium (HWE); however, several markers showed significant deviations, likely related to the presence of null alleles or a heterozygote deficit. Overall, the obtained results indicate a high level of genetic variability in the studied population and demonstrate the strong suitability of the analyzed marker set for studies of genetic diversity and population structure.

For some loci, namely CMS3, LCA23, YWLL08, YWLL59, LCA68, VOLP04, VOLP03, and YWLL43x, deviations from HWE were observed. This observation is supported by the F(null) index, which was low for most loci; however, for some markers it was clearly elevated (YWLL59 = 0.431, LCA68 = 0.313, YWLL43X = 0.152), suggesting a possible influence of null alleles and the need for cautious interpretation of these loci.

### 3.4. Population Structure

Monomorphic markers and markers with an F(null) coefficient > 0.045 were excluded from further analyses. Analyses were conducted using 37 markers after excluding YWLL59, LCA68, YWLL43X, CMS3, LCA23, CMS104, LCA90, YWLL02, and SRY.

**Figure 1:**
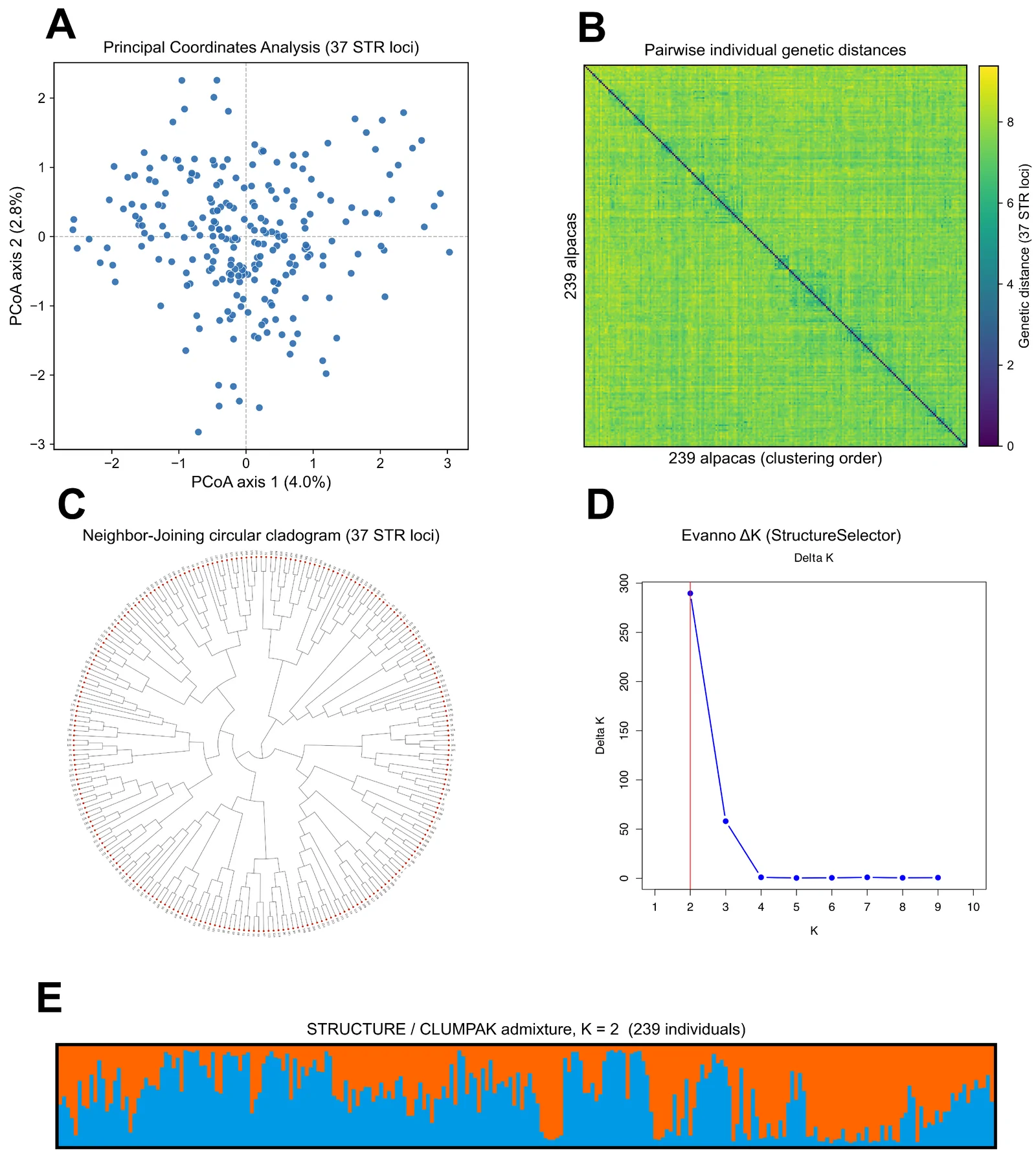
Genetic structure in 239 Huacaya alpacas. (**A**) Principal coordinates analysis (PCoA); (**B**) heatmap of pairwise genetic distances; (**C**) Neighbor-Joining circular cladogram; (**D**) Evanno DeltaK plot (StructureSelector) peaking at K = 2; (**E**) STRUCTURE for K = 2.

Principal Coordinates Analysis (PCoA) (Figure 1A) did not reveal any population substructure. Individuals formed a single major cluster, while the first two axes explained only 4.0% and 2.8% of the total genetic variation, indicating that the studied alpaca population appears to be genetically homogeneous and lacks detectable internal genetic differentiation.

The heatmap of the genetic distance matrix (Figure 1B) likewise revealed no distinct groupings. Instead, it displayed a relatively uniform color pattern, with no discernible blocks along the diagonal, indicating the absence of cryptic subgroups. This finding further supports the genetic homogeneity of the alpaca population bred in Poland and is consistent with random mating. The Neighbor-Joining (NJ) cladogram (Figure 1C) revealed no deeply divergent, well-defined clades and showed no hierarchical population structure.

Bayesian clustering analysis likewise failed to reveal any distinct genetic structure within the population. The Evanno method identified a maximum ΔK value at K = 2; however, this result should be interpreted with caution, as the membership coefficient plot for K = 2 did not indicate any clear subdivision. Most individuals exhibited intermediate and admixed membership coefficients, suggesting a high degree of genetic overlap between the two inferred clusters (Figure 1D,E).

## 4. Discussion

In this study, a genetic sex identification marker for alpacas was developed, along with three microsatellite panels. Their utility was then evaluated for individual identification and parentage testing, as well as for assessing the genetic structure of the Huacaya alpaca population bred in Poland.

### 4.1. Sex Determination Marker, Individual Identification and Parentage Testing

Alpacas do not exhibit pronounced sexual dimorphism: females and males typically reach a height of approximately 1 m and differ mainly in body weight. Females usually weigh between 50 and 70 kg, whereas males range from 60 to 90 kg [10]. Due to the lack of clear differences in external appearance, the sex of alpacas is primarily determined based on their reproductive organs. On the other hand, sex can also be determined genetically. This enables monitoring the accuracy of data provided by breeders, as well as confirming the correct labeling and processing of samples within the laboratory. It serves as an internal control, which is of considerable importance from a laboratory perspective [41].

Sex identification is widely implemented in microsatellite panels recommended by the International Society for Animal Genetics (ISAG) across multiple animal species [22]. Moreover, laboratories are frequently requested to determine the sex of animals in forensic cases involving non-human biological evidence [42]. Detection of the SRY sequence enables differentiation between DNA samples originating from males and those originating from females, as female DNA does not contain this gene. In forensic analyses, the detection of the SRY gene may serve as confirmation of male sex determination [42,43].

The parameters of individual identification and parentage testing obtained for the 44 markers demonstrated their high utility for these types of analyses. The exclusion probabilities for the first parent, second parent, parent pair, identity, and sibling identity exceeded 99.99%. Moreover, the extremely low probability of identity non-exclusion (2.178 × 10^−50^) indicates a very high power of individual identification, while the value obtained for sibling identity (1.125 × 10^−18^) confirms the high discriminatory power of the panel, even among closely related individuals [44].

A marker is generally considered informative when its PIC value exceeds 0.5 [45]. In Panel I, all markers exhibited PIC values above 0.5, with the exception of YWLL02 and SRY (monomorphic). In Panel II, three markers (CMS3, P194, and YWLL46) showed PIC values below 0.5, while in Panel III only the CMS104 marker had a PIC value below this threshold. Nevertheless, the mean PIC values observed in the present study were similar to those previously reported for other alpaca populations [4,12,13,46,47].

Moreover, each of the analyzed panels evaluated separately demonstrated a higher exclusion probability than the panel developed for alpacas and described by Agapito et al. [46]. These results confirm that all three analyzed panels exhibit high efficiency for parentage analysis and individual identification. The obtained results indicate that the developed set of markers can be successfully applied both in population genetic studies and in routine pedigree control of alpacas.

The use of more than one microsatellite panel is required in cases of questionable results, for example those resulting from polymerase slippage or the presence of null alleles [48]. High values of null allele frequency were observed for the YWLL59 marker (Panel II), CMS3 (Panel II), LCA68 marker (Panel III), and YWLL43X marker (Panel III). Previous studies conducted on the Polish alpaca population also reported a high value for the YWLL43X marker [4]. However, this marker remains on the list of additional microsatellites recommended by ISAG for alpacas [49]; therefore, it was included in Panel III despite its high F(null) coefficient. Null alleles represent a challenge in parentage analysis because they may cause mismatches within a single locus. In such cases, the offspring is homozygous at a given locus, and one of the parents also appears to be homozygous; however, the allele detected in the offspring differs from the allele observed in the parent. This results in the presence of two different homozygous genotypes at the same locus, which may complicate accurate pedigree verification [48,50,51].

It should also be noted that, in addition to the presence of null alleles, a particularly common problem associated with microsatellite markers is allelic dropout, in which one of the two alleles present in a diploid genotype fails to amplify during PCR, resulting in the incorrect identification of a heterozygote as a homozygote. Unfortunately, the use of a greater number of highly polymorphic markers is often associated with an increased genotyping error rate in multilocus genotypes. Typing errors may cause apparent mismatches between parental and offspring genotypes and, consequently, lead to false exclusions of parentage if they are not properly accounted for in the analysis [52].

### 4.2. Genetic Diversity and Population Structure

Maintaining a high level of genetic diversity is a fundamental objective of genetic resource conservation programs. This diversity is essential for enabling populations to adapt to future environmental changes and ensuring their long-term capacity to respond to both natural and artificial selection [11].

Previous studies on alpacas kept in Poland, involving analyses based on microsatellite markers [4] and mitochondrial DNA [17], revealed a relatively high level of genetic diversity. Our results confirmed that the genetic diversity of alpacas in Poland remains high. The obtained results did not indicate substantial inbreeding within the analyzed population.

The high mean Na value, together with the mean Ne value, indicates that the observed variability is not attributable merely to rare alleles but rather reflects true genetic diversity within the population [53]. High values of the Shannon index reflect high genetic diversity, arising from both high allelic richness and a relatively even distribution of allele frequencies within the population [54]. The Ho was slightly lower than the He, suggesting a modest heterozygote deficit that may reflect both biological processes (e.g., population structure) and methodological factors such as null alleles or genotyping errors [55]. Deviations from HWE may result from a variety of biological and methodological factors. The most commonly reported include selection, population structure, small sample size, low marker polymorphism, the presence of null alleles, and selection favoring homozygous individuals. All of these factors may contribute to a reduction in the frequency of heterozygotes within a population. Differences in HWE results among alpaca populations worldwide [14,15,46,47,56] and even in Poland [4] suggest that the behavior of individual markers may depend on the specific characteristics of the population under investigation as well as the breeding program being implemented [47].

The observed genetic diversity suggests that breeding practices currently applied in Poland have been sufficient to maintain a broad genetic background. Peralta et al. (2025) [11] showed that closed populations subjected to genetic improvement programs targeting reproductive traits and fiber quality exhibited a substantial reduction in genetic diversity compared with populations maintained by local communities, where mating patterns are more random. In Poland, alpacas are also mainly bred for the production of high-quality fiber; nevertheless, breeders ensure the implementation of appropriate mating systems to maintain genetic diversity within the population [4]. In community-based breeding systems, in which herds are managed independently by local breeders and mating decisions are often made without centralized coordination, the exchange of animals among herds contributes to the maintenance of a broad gene pool, thereby increasing the population’s adaptive capacity and resilience [11].

Moreover, the reliability of our results is supported by the relatively large number of genetic markers (44) used to assess genetic diversity. Previous studies on South American camelids have indicated that the use of a limited number of markers or a small sample size may compromise the accuracy and representativeness of genetic diversity estimates [11].

The analyzed alpaca population appeared genetically homogeneous, with no evidence of distinct genetic clusters. No detectable population substructure or distinct genetic lineages, family groups, or groups of common origin were identified. Comparable findings have been reported in Peru, particularly in the Quimsachata population, which functions as a genetic resource bank for alpacas, and in the Ajoyani population, where breeding efforts are primarily focused on maintaining white-fleeced Huacaya alpacas [12].

In contrast, Peralta’s et al. (2025) [11] study of Peruvian alpacas identified three distinct genetic clusters, reflecting the influence of breeding management practices and gene flow in the Peruvian Andes. Furthermore, the Bolivian alpaca population also did not exhibit a panmictic structure, with five distinct subpopulations identified [15]. Peru and Bolivia are considered the native regions of alpaca distribution, with Peru hosting the largest alpaca population worldwide (1.5 mln) [57] and Bolivia ranking second, with approximately 460 k individuals [15]. These regions overlap with the historical territory of the Inca Empire, which reached its peak between the 12th and 16th centuries. Archeological evidence suggests that the Incas were highly advanced alpaca breeders, and the quality of alpaca fiber was regarded as comparable to that of vicuña fiber, often referred to as the “gold of the Andes” [1,58]. This historical continuity may explain the genetic structure observed in South American alpaca populations, which share a common evolutionary and breeding history. In contrast, alpaca breeding in Poland has a much shorter history, beginning only in 2004 [4]. The observed differences may be explained by the distinct breeding objectives pursued in different regions of the world and across different alpaca populations, thereby shaping their genetic structure [13].

## 5. Conclusions

This study demonstrated that the developed genetic sex identification marker and three microsatellite panels represent effective tools for genetic analyses of Huacaya alpacas bred in Poland. The SRY-based sex identification marker enables reliable molecular sex determination and may serve as an internal quality control tool for verifying information provided by breeders and supporting laboratory sample processing. The set of 44 microsatellite markers showed high efficiency in individual identification and parentage analysis, with very high exclusion probabilities and strong discriminatory power. The developed panels can be successfully applied both in population genetic studies and in routine pedigree verification of alpacas.

Interestingly, the analyzed alpaca population in Poland maintains a high level of genetic diversity. The obtained results do not indicate a substantial level of inbreeding and suggest that current breeding practices may be sufficient to preserve a broad genetic background. Moreover, the analyses did not reveal clear evidence of genetic substructure, distinct subpopulations, or separate genetic lineages. Maintaining current breeding strategies and monitoring genetic diversity will be crucial for supporting the long-term stability and adaptive potential of the alpaca population in Poland.

## Figures and Tables

**Table 1:** Characteristics of 46 markers.

Locus	Primer (5′-3′) Forward	Primer (5′-3′) Reverse	Dye	Size Range (bp)	Multiplex	Reference
LCA19	TAAGTCCAGCCCCACACTCA	GGTGAAGGGGCTTGATCTTC	6-FAM	80–140	Panel I	[23]
LCA65	TTTTTCCCCTGTGGTTGAAT	AACTCAGCTGTTGTCAGGGG	6-FAM	160–200		[24]
LCA66	GTGCAGCGTCCAAATAGTCA	CCAGCATCGTCCAGTATTCA	6-FAM	210–260		[24]
YWLL02	GTGCACTCAGATACCTTCACA	TACATCTGCAATGATCGACCC	6-FAM	300–325		[25]
VOLP05	ACTTAATCACCTGGATGTAT	ATATGGTTCACTGTGTTTACT	VIC	130–170		[26]
LCA5	GTGGTTTTTGCCCAAGCTC	ACCTCCAGTCTGGGGATTTC	VIC	180–215		[23]
LGU91	GTCTCCGGTTTGCAGATGAG	AGAAGCAGAATGGTAGTGAAGAA	VIC	220–250		[27]
LCA99	CAGGTATCAGGAGACGGGCT	AGCATTTATCAAGGAACACCAGC	VIC	260–300		[28]
LCA24	ACTCACGGGTGACATACACTG	GAGCAGTGTTTGGTTTGCATT	NED	100–125		[23]
LCA56	ATGGTGTTTACAGGGCGTTG	GCATTACTGAAAAGCCCAGG	NED	130–170		[24]
LGU50	CTGCTGTGCTTGTCACCCTA	AGCACCACATGCCTCTAAGT	NED	180–200		[27]
LCA8	GCTGAACCACAATGCAAAGA	AATGCAGATGTGCCTCAGTT	NED	220–265		[23]
SRY	GGCTCTAGAGATCCCAAAATGC	TAGTCTCTGTGCCTCCTCGA	PET	110–120		Own marker
LCA37	AAACCTAATTACCTCCCCCA	CCATGTAGTTGCAGGACACG	PET	125–185		[23]
LCA94	GTCCATTCATCCAGCACAGG	ACATTTGGCAATCTCTGGAGAA	PET	190–210		[28]
LGU49	TCTAGGTCCATCCCTGTTGC	GTGCTGGAATAGTGCCCAGT	PET	215–255		[27]
P132	CAGAGGAGGGACCACTAATGCTGGC	GGGGCAAGTGAAGTGAGTGAAATGG	6-FAM	80–110	Panel II	[29]
LCA23	TCACGGCAAAGACGTGAATA	CCAGGAAACACACACCCAC	6-FAM	125–172		[23]
YWL40	CACATGACCATGTCCCCTTAT	CCAGTGACAGTGTGACTAAGA	6-FAM	175–200		[25]
YWLL29	GAAGGCAGGAGAAAAGGTAG	CAGAGGCTTAATAACTTGCAG	6-FAM	210–235		[25]
CMS13	TAGCCTGACTCTATCCATTTCTC	ATTATTTGGAATTCAACTGTAAGG	6-FAM	240–270		[30]
YWLL44	CTCAACAATGCTAGACCTTGG	GAGAACACAGGCTGGTGAATA	VIC	85–130		[25]
P194	AGCAGGTGAAAAGCAGAATTGTGTG	AGTTTTTCCATTGCCGTTGTCAGAG	VIC	140–160		[29]
YWLL38	GGCCTAAATCCTACTAGAC	CCTCTCACTCTTGTTCTCCTC	VIC	170–185		[25]
LCA18	TCCACCCATTTAGACACAAGC	TAGGAAGCTCCAAGAAGAAAAGAC	VIC	220–248		[23]
CMS3	GCTCTCTGTGCCTCAATATG	ACCATAGTTCTGGGTAGCA	VIC	250–290		[30]
YWL46	AAGCAGAGTGATTTAACCGTG	GGATGACTAAGACTGCTCTGA	NED	90–115		[25]
YWLL08	ATCAAGTTTGAGGTGCTTTCC	CCATGGCATTGTGTTGAAGAC	NED	120–195		[25]
LGU68	CATCTACATGCCCCTGTGTG	TGCAGGGAGGACTAACAGGT	NED	205–240		[27]
YWLL59	TGTGCAGGAGTTAGGTGTA	CCATGTCTCTGAAGCTCTGGA	PET	90–137		[25]
YWLL36	AGTCTTGGTGTGGTGGTAGA A	TGCCAGGATACTGACAGTGAT	PET	138–80		[25]
PCTD17	CCCTCTCACCTGTCTACTTG	GTATTCTGGCATTGGTTTGT	PET	188–225		[29]
LCA77	TGTTGACTAGAGCCTTTTCTTCTTT	GGGCAAGAGAGACTGACTGG	PET	235–270		[24]
CMS104	TTAGGTCCCTGGGCTTTATG	AAATTGTGCATTCTCTTG CATC	6-FAM	80–110	Panel III	[30]
CMS121	CAAGAGAACTGGTGA GGATTTTC	AGTTGATAAAAATAC AGCTGGAAAG	6-FAM	125–170		[30]
LGU93	CCTGTGGAGCTAGGCCTTC	TTTGTAATCCCAGCCTGCTC	6-FAM	180–214		[27]
LGU76	TTCCTTCCATTGAAGCAGGT	TGAGATGCACTGCTTTGGATA	6-FAM	216–270		[27]
YWLL43-X	ATACCTCTCTTGCTCTCTCTC	CCTCTACAACCATGTTAG CCA	VIC	120–160		[25]
LGU52	GTGGTTGGAACCTGCAACTC	TTGGGACCTGCTTCCATAAC	VIC	180–210		[27]
VOLP04	GCATTTCTCCGTAATCATTG	TGACACCTTTTGTTTCCATT	VIC	220–260		[31]
VOLP03	AGACGGTTGGGAAGG TGG	CGACAGCAAGGCACA GGA	NED	125–175		[26]
VOLP32	GTGATCGGAATGGCTTGAAA	CAGCGAGCACCTGAAAGAA	NED	184–249		[26]
CVRL07	AATACCCTAGTTGAAGCTCTGTCCT	GAGTGCCTTTATAAATATGGGTCTG	NED	250–285		[32]
CMS25	GAT CCT CCT GCG TTC TTA TT	CTA GCC TTT GAT TGG AGC AT	PET	95–140		[30]
LCA68	TCCTGTCTGTGAGAAGGCTG	CCGAAGGAAAAATAAAATGGAA	PET	185–215		[28]
LCA90	TATAACCCTGGTCTCGCCAA	CCAAGTAGTATTCCATTATGCG	PET	225–265		[28]

**Table 2:** Genetic diversity parameters and parentage assignment statistics for 14 microsatellite loci (Multiplex panel I) in Huacaya alpacas.

Locus	Na	Ho	He	PIC	NE-1P	NE-2P	NE-PP	NE-I	NE-SI	F(Null)
LCA19	15	0.720	0.727	0.699	0.655	0.471	0.268	0.103	0.412	0.0091
LCA65	13	0.870	0.870	0.856	0.417	0.262	0.103	0.031	0.323	−0.0005
LCA66	15	0.808	0.828	0.813	0.487	0.318	0.134	0.044	0.347	0.0114
VOLP05	10	0.732	0.786	0.758	0.584	0.405	0.217	0.074	0.375	0.0364
LCA5	11	0.787	0.765	0.730	0.625	0.447	0.261	0.090	0.390	−0.0144
LCA99	12	0.808	0.838	0.819	0.485	0.318	0.144	0.045	0.342	0.0184
LGU91	7	0.577	0.610	0.572	0.787	0.613	0.422	0.190	0.492	0.0245
LCA24	8	0.711	0.702	0.660	0.703	0.526	0.336	0.130	0.432	−0.0069
LCA56	12	0.828	0.804	0.779	0.552	0.376	0.191	0.063	0.364	−0.0169
LGU50	5	0.724	0.690	0.634	0.735	0.569	0.395	0.152	0.443	−0.0257
LCA8	12	0.866	0.853	0.836	0.458	0.294	0.127	0.039	0.333	−0.0082
LCA37	16	0.820	0.860	0.846	0.431	0.274	0.109	0.034	0.328	0.0237
LCA94	7	0.791	0.814	0.788	0.546	0.370	0.192	0.061	0.358	0.0132
LGU49	13	0.845	0.860	0.845	0.439	0.279	0.115	0.035	0.329	0.0081
Mean	11.143	0.778	0.786	0.764	0.554	0.402	0.214	0.078	0.370	0.0072

Na, number of alleles; Ho, observed heterozygosity; He, expected heterozygosity; PIC, polymorphic information content; NE-1P, non-exclusion probability for the first parent; NE-2P, non-exclusion probability for the second parent; NE-PP, non-exclusion probability for a parent pair; NE-I, non-exclusion probability of identity; NE-SI, non-exclusion probability of sibling identity; F(Null), estimated null allele frequency.

**Table 3:** Genetic diversity parameters and parentage assignment statistics for 17 microsatellite loci (Multiplex panel II) in Huacaya alpacas.

Locus	Na	Ho	He	PIC	NE-1P	NE-2P	NE-PP	NE-I	NE-SI	F(Null)
YWLL40	6	0.669	0.701	0.648	0.721	0.553	0.378	0.143	0.435	0.0188
YWLL29	9	0.690	0.719	0.688	0.671	0.487	0.286	0.110	0.418	0.0246
CMS13	11	0.778	0.771	0.745	0.601	0.419	0.223	0.079	0.384	−0.0078
LCA23	15	0.723	0.852	0.836	0.457	0.294	0.126	0.039	0.334	0.0851
P132	4	0.661	0.665	0.591	0.778	0.630	0.480	0.186	0.464	0.0037
YWLL44	16	0.849	0.873	0.860	0.408	0.255	0.098	0.029	0.321	0.0140
YWLL38	5	0.661	0.701	0.654	0.715	0.541	0.358	0.136	0.434	0.0368
CMS3	2	0.353	0.500	0.375	0.875	0.813	0.719	0.375	0.594	0.1724
P194	4	0.569	0.557	0.472	0.844	0.725	0.587	0.281	0.542	−0.0128
LCA18	9	0.795	0.753	0.729	0.619	0.434	0.232	0.085	0.395	−0.0353
YWLL46	5	0.347	0.378	0.339	0.927	0.809	0.686	0.426	0.668	0.0404
YWLL08	29	0.925	0.936	0.932	0.228	0.129	0.028	0.008	0.284	0.0054
LGU68	9	0.586	0.577	0.547	0.806	0.630	0.433	0.209	0.514	−0.0194
PCTD17	8	0.661	0.694	0.651	0.714	0.538	0.349	0.136	0.437	0.0264
YWLL59	16	0.351	0.880	0.870	0.383	0.236	0.084	0.025	0.316	0.4310
YWLL36	14	0.841	0.879	0.867	0.393	0.243	0.090	0.026	0.317	0.0229
LCA77	12	0.707	0.765	0.734	0.617	0.437	0.245	0.086	0.389	0.0395
Mean	10.235	0.657	0.718	0.691	0.648	0.490	0.337	0.144	0.427	0.0596

Na, number of alleles; Ho, observed heterozygosity; He, expected heterozygosity; PIC, polymorphic information content; NE-1P, non-exclusion probability for the first parent; NE-2P, non-exclusion probability for the second parent; NE-PP, non-exclusion probability for a parent pair; NE-I, non-exclusion probability of identity; NE-SI, non-exclusion probability of sibling identity; F(Null), estimated null allele frequency.

**Table 4:** Genetic diversity parameters and parentage assignment statistics for 13 microsatellite loci (Multiplex panel III) in Huacaya alpacas.

Locus	Na	Ho	He	PIC	NE-1P	NE-2P	NE-PP	NE-I	NE-SI	F(Null)
CMS104	4	0.364	0.439	0.389	0.903	0.778	0.646	0.365	0.622	0.0998
CMS121	12	0.816	0.832	0.812	0.501	0.331	0.155	0.048	0.346	0.0086
LGU93	13	0.820	0.861	0.846	0.437	0.277	0.114	0.034	0.328	0.0260
LGU76	15	0.803	0.839	0.822	0.479	0.312	0.136	0.043	0.341	0.0199
LGU52	9	0.678	0.703	0.670	0.693	0.509	0.309	0.121	0.429	0.0128
VOLP04	22	0.824	0.853	0.841	0.436	0.277	0.106	0.034	0.332	0.0174
VOLP03	10	0.736	0.765	0.737	0.614	0.432	0.238	0.084	0.388	0.0154
VOLP32	23	0.862	0.882	0.872	0.375	0.231	0.079	0.024	0.315	0.0114
CVRL07	17	0.833	0.875	0.862	0.404	0.252	0.096	0.028	0.320	0.0256
CMS25	10	0.732	0.711	0.671	0.693	0.515	0.324	0.124	0.425	−0.0218
LCA68	14	0.418	0.803	0.777	0.556	0.380	0.196	0.065	0.365	0.3134
LCA90	15	0.741	0.812	0.790	0.531	0.357	0.172	0.057	0.358	0.0483
YWLL43X	10	0.477	0.655	0.602	0.749	0.586	0.400	0.172	0.466	0.1519
Mean	13.385	0.700	0.772	0.739	0.575	0.428	0.253	0.101	0.378	0.0522

Na, number of alleles; Ho, observed heterozygosity; He, expected heterozygosity; PIC, polymorphic information content; NE-1P, non-exclusion probability for the first parent; NE-2P, non-exclusion probability for the second parent; NE-PP, non-exclusion probability for a parent pair; NE-I, non-exclusion probability of identity; NE-SI, non-exclusion probability of sibling identity; F(Null), estimated null allele frequency.

**Table 5:** Combined non-exclusion probabilities (NEs) and corresponding exclusion probabilities for parentage and identity analysis for all 44 markers.

Parameter	Combined Non-Exclusion Probability (NE)	Exclusion Probability (1 − NE)	Exclusion Probability (%)
First parent (NE-1P)	1.584 × 10^−11^	0.99999999998416	99.99
Second parent (NE-2P)	2.720 × 10^−18^	0.99999999999999999728	99.99
Parent pair (NE-PP)	1.081 × 10^−30^	0.999999999999999999999999999999	99.99
Identity (NE-I)	2.178 × 10^−50^	0.99999999999999999999999999999999999999999999999998	99.99
Sibling identity (NE-SI)	1.125 × 10^−18^	0.999999999999999998875	99.99

NE-1P, non-exclusion probability for the first parent; NE-2P, non-exclusion probability for the second parent; NE-PP, non-exclusion probability for the parent pair; NE-I, non-exclusion probability for identity; NE-SI, non-exclusion probability for sibling identity.

**Table 6:** Genetic diversity and summary of chi-square tests for Hardy–Weinberg equilibrium for all 44 markers.

Locus	Na	Ne	I	Ho	He	F	PIC	F(Null)	DF	ChiSq	*p*	HWE
LCA19	15	3.6683	1.7051	0.7197	0.7274	0.0106	0.699	0.0091	105	165.3013	0.0002	***
LCA65	13	7.6884	2.1933	0.8703	0.8699	−0.0004	0.856	−0.0005	78	75.6135	0.5555	ns
LCA66	15	5.807	2.1555	0.8075	0.8278	0.0245	0.813	0.0114	105	115.4089	0.2292	ns
VOLP05	10	4.6834	1.7755	0.7322	0.7865	0.069	0.758	0.0364	45	45.7057	0.4426	ns
LCA5	11	4.2585	1.6646	0.7866	0.7652	−0.028	0.730	−0.0144	55	29.9234	0.9977	ns
LCA99	12	6.1873	2.0509	0.8075	0.8384	0.0368	0.819	0.0184	66	90.3396	0.025	*
LGU91	7	2.5657	1.2452	0.5774	0.6102	0.0538	0.572	0.0245	21	32.9304	0.047	*
LCA24	8	3.3511	1.4721	0.7113	0.7016	−0.0138	0.660	−0.0069	28	13.6273	0.9896	ns
LCA56	12	5.091	1.8879	0.8285	0.8036	−0.031	0.779	−0.0169	66	69.337	0.3656	ns
LGU50	5	3.2219	1.2906	0.7238	0.6896	−0.0496	0.634	−0.0257	10	19.3187	0.0364	*
LCA8	12	6.788	2.0797	0.8661	0.8527	−0.0157	0.836	−0.0082	66	41.9007	0.991	ns
LCA37	16	7.1392	2.2506	0.8201	0.8599	0.0463	0.846	0.0237	120	161.9341	0.0065	**
LCA94	7	5.3832	1.7775	0.7908	0.8142	0.0288	0.788	0.0132	21	22.1941	0.3884	ns
LGU49	13	7.1237	2.1525	0.8452	0.8596	0.0168	0.845	0.0081	78	70.8475	0.7045	ns
YWLL40	6	3.3464	1.3477	0.6695	0.7012	0.0452	0.648	0.0188	15	17.359	0.2979	ns
YWLL29	9	3.5578	1.5839	0.6904	0.7189	0.0397	0.688	0.0246	36	24.8624	0.919	ns
CMS13	11	4.3581	1.7524	0.7782	0.7705	−0.01	0.745	−0.0078	55	49.4582	0.6855	ns
LCA23	15	6.7675	2.1137	0.7227	0.8522	0.152	0.836	0.0851	105	711.4268	0	***
P132	4	2.9821	1.1063	0.6611	0.6647	0.0054	0.591	0.0037	6	4.1634	0.6546	ns
YWLL44	16	7.888	2.2325	0.8494	0.8732	0.0273	0.860	0.0140	120	108.5462	0.7645	ns
YWLL38	5	3.3407	1.3502	0.6611	0.7007	0.0565	0.654	0.0368	10	20.772	0.0227	*
CMS3	2	2.000	0.6931	0.3529	0.5	0.2941	0.375	0.1724	1	19.1158	0	***
P194	4	2.2564	0.9243	0.569	0.5568	−0.0219	0.472	−0.0128	6	239.6334	0	***
LCA18	9	4.0536	1.7186	0.795	0.7533	−0.0553	0.729	−0.0353	36	43.0789	0.1942	ns
YWLL46	5	1.6068	0.7213	0.3473	0.3776	0.0804	0.339	0.0404	10	7.8507	0.6434	ns
YWLL08	29	15.5898	2.9868	0.9247	0.9359	0.0119	0.932	0.0054	406	853.9434	0	***
LGU68	9	2.3665	1.2536	0.5858	0.5774	−0.0144	0.547	−0.0194	36	21.2389	0.9759	ns
PCTD17	8	3.2673	1.4315	0.6611	0.6939	0.0473	0.651	0.0264	28	16.8542	0.9514	ns
YWLL59	16	8.3614	2.3607	0.3515	0.8804	0.6008	0.870	0.4310	120	1337.9587	0	***
YWLL36	14	8.2658	2.2799	0.841	0.879	0.0432	0.867	0.0229	91	82.5377	0.7251	ns
LCA77	12	4.2472	1.7135	0.7071	0.7646	0.0751	0.734	0.0395	66	76.9036	0.1689	ns
CMS104	4	1.7833	0.7778	0.364	0.4392	0.1713	0.389	0.0998	6	15.8354	0.0147	*
CMS121	12	5.97	1.9607	0.8159	0.8325	0.0199	0.812	0.0086	66	113.6414	0.0002	***
LGU93	13	7.1905	2.1514	0.8201	0.8609	0.0474	0.846	0.0260	78	84.4435	0.2893	ns
LGU76	15	6.1923	2.0733	0.8033	0.8385	0.0419	0.822	0.0199	105	140.5985	0.0117	*
LGU52	9	3.3621	1.522	0.6778	0.7026	0.0352	0.670	0.0128	36	50.4195	0.0559	ns
VOLP04	22	6.8127	2.3511	0.8243	0.8532	0.0339	0.841	0.0174	231	330.1427	0	***
VOLP03	10	4.2623	1.6816	0.7364	0.7654	0.0379	0.737	0.0154	45	154.0879	0	***
VOLP32	23	8.4499	2.4682	0.8619	0.8817	0.0224	0.872	0.0114	253	288.6644	0.0611	ns
CVRL07	17	7.9817	2.2624	0.8326	0.8747	0.0481	0.862	0.0256	136	155.7168	0.1186	ns
CMS25	10	3.4636	1.4954	0.7322	0.7113	−0.0294	0.671	−0.0218	45	42.5045	0.5782	ns
LCA68	14	5.0718	1.878	0.4184	0.8028	0.4788	0.777	0.3134	91	720.4069	0	***
LCA90	15	5.3314	1.9794	0.7406	0.8124	0.0884	0.790	0.0483	105	180.011	0	***
YWLL43X	10	2.8971	1.4106	0.477	0.6548	0.2716	0.602	0.1519	45	302.2381	0	***
Mean	11.4545	5.1359	1.7564	0.7082	0.7554	0.0635	0.727	0.0347				

Na, number of observed alleles; Ne, effective number of alleles; I, Shannon’s information index; Ho, observed heterozygosity; He, expected heterozygosity; F, inbreeding coefficient; PIC, polymorphic information content; F(Null), estimated frequency of null alleles; DF, degrees of freedom; ChiSq, chi-square test statistic; *p*, significance level of the HWE test; * *p* < 0.05, ** *p* < 0.01, *** *p* < 0.001; ns, not significant. HWE was tested separately for each locus and population by comparing the observed genotype frequencies with those expected from the observed allele frequencies under HWE (p^2^, 2pq, q^2^) using a chi-square test. Therefore, the HWE values presented in Table represent a test for departure from HWE and do not constitute an assumption imposed on the calculation of other genetic diversity parameters.

## Data Availability

The data supporting the findings of this study are not publicly available but are available from the corresponding author upon reasonable request.

## Supplementary Materials

The following supporting information can be downloaded at https://www.mdpi.com/article/10.3390/biology15171555/s1, Table S1: Thermal cycling conditions for three microsatellite panels; Table S2. A summary of genetic markers included in the three marker panels and their use in genetic diversity and population structure analyses; Table S3: Combined non-exclusion probabilities (NEs) and corresponding exclusion probabilities for parentage and identity analysis for Multiplex panel I; Table S4: Combined non-exclusion probabilities (NEs) and corresponding exclusion probabilities for parentage and identity analysis for Multiplex panel II; Table S5: Combined non-exclusion probabilities (NEs) and corresponding exclusion probabilities for parentage and identity analysis for Multiplex panel III.
