# Supplementary material for: Genetic Characterization of Huacaya Alpacas (Vicugna pacos) in Poland Using Microsatellite Markers
Source: Biology (Basel). 2026 Sep 5;15(17):1555. doi: 10.3390/biology15171555 (PMC13565558; doi:10.3390/biology15171555)
Supplement: Supplementary file 1 [file biology-15-01555-s001.zip › biology-4507317-supplementary.pdf]

Table S1. Thermal cycling conditions for three microsatellite panels.

Step	Temperature [°C]	Time [min]	Number of cycles
Initial denaturation	95	5	1
Denaturation	95	0.5	
Annealing	60	1.5	28
Extension	72	0.5	
Final extension	60	30	1

Table S2. Summary of genetic markers included in the three marker panels and their use in genetic diversity and population structure analyses.

Panel	No. of markers	Markers excluded from genetic diversity analysis	Markers used for genetic diversity analysis	Markers excluded from population structure analysis	Markers used for population structure analysis
Panel I	16	YWLL02, SRY	14	YWLL02, SRY	14
Panel II	17	None	17	YWLL59, CMS3, LCA23	14
Panel III	13	None	13	LCA68, YWLL43X, CMS104, LCA90	9
Total	46	2	44	9	37

Table S3. Combined non-exclusion probabilities (NEs) and corresponding exclusion probabilities for parentage and identity analysis for Multiplex panel I.

Parameter	Combined non-exclusion probability (NE)	Exclusion probability (1 − NE)	Exclusion probability (%)
First parent (NE-1P)	2.4990×10^{-4}	0.99975010	99.97
Second parent (NE-2P)	1.300×10^{-6}	0.99999870	99.99
Parent pair (NE-PP)	9.908×10^{-11}	0.99999999990092	99.99
Identity (NE-I)	2.777×10^{-17}	0.99999999999999997223	99.99
Sibling identity (NE-SI)	1.02×10^{-6}	0.99999898	99.99

NE-1P, non-exclusion probability for the first parent; NE-2P, non-exclusion probability for the second parent; NE-PP, non-exclusion probability for the parent pair; NE-I, non-exclusion probability for identity; NE-SI, non-exclusion probability for sibling identity.

Table S4. Combined non-exclusion probabilities (NEs) and corresponding exclusion probabilities for parentage and identity analysis for Multiplex panel II.

Parameter	Combined non-exclusion probability (NE)	Exclusion probability (1 − NE)	Exclusion probability (%)
First parent (NE-1P)	1.5570×10^{-4}	0.99984430	99.98
Second parent (NE-2P)	6.50×10^{-7}	0.99999935	99.99
Parent pair (NE-PP)	2.721×10^{-11}	0.99999999997279	99.99
Identity (NE-I)	1.653×10^{-18}	0.999999999999999998347	99.99
Sibling identity (NE-SI)	3.20×10^{-7}	0.99999968	99.99

NE-1P, non-exclusion probability for the first parent; NE-2P, non-exclusion probability for the second parent; NE-PP, non-exclusion probability for the parent pair; NE-I, non-exclusion probability for identity; NE-SI, non-exclusion probability for sibling identity.

Table S5. Combined non-exclusion probabilities (NEs) and corresponding exclusion probabilities for parentage and identity analysis Multiplex panel III.

Parameter	Combined non-exclusion probability (NE)	Exclusion probability (1 − NE)	Exclusion probability (%)
First parent (NE-1P)	4.072×10^{-4}	0.99959284	99.96
Second parent (NE-2P)	3.22×10^{-6}	0.99999678	99.99
Parent pair (NE-PP)	4.012×10^{-10}	0.9999999995988	99.99
Identity (NE-I)	4.746×10^{-16}	0.9999999999999995254	99.99
Sibling identity (NE-SI)	3.47×10^{-6}	0.99999653	99.93

NE-1P, non-exclusion probability for the first parent; NE-2P, non-exclusion probability for the second parent; NE-PP, non-exclusion probability for the parent pair; NE-I, non-exclusion probability for identity; NE-SI, non-exclusion probability for sibling identity.
